# The Effect of the Addition of Waste-Derived Biofillers on the Degradation of Ethylene–Norbornene (EN) Copolymers Under Laboratory Composting Conditions

**DOI:** 10.3390/polym17111483

**Published:** 2025-05-27

**Authors:** Malgorzata Latos-Brozio, Michał Bocianowski, Magdalena Efenberger-Szmechtyk, Małgorzata Piotrowska, Anna Masek

**Affiliations:** 1 Institute of Polymer and Dye Technology, Faculty of Chemistry, Lodz University of Technology, Stefanowskiego 16, 90-537 Lodz, Poland; 2 Institute of Fermentation Technology and Microbiology, Faculty of Biotechnology and Food Sciences, Lodz University of Technology, Wólczańska 71/173, 90-537 Lodz, Poland; magdalena.efenberger-szmechtyk@p.lodz.pl (M.E.-S.); malgorzata.piotrowska@p.lodz.pl (M.P.)

**Keywords:** biofillers, composting, ethylene–norbornene copolymer

## Abstract

The objective of the present study was to investigate the potential effects of biofillers derived from fruit waste, a byproduct of the food-processing industry, on the degradation of ethylene–norbornene (EN) copolymers under the controlled conditions of laboratory composting. This manuscript provides a comprehensive analysis of the influence of waste biofillers on the biological degradation of EN-based materials, thereby filling a gap in the existing literature on the subject. The concept of this work encompasses the enhancement of the degradability of synthetic EN through the incorporation of bioadditives. Waste apple and chokeberry pomace were added to EN as biofillers in amounts of 5, 10, and 15 phr (parts per hundred rubber). The polymeric materials were composted for 3 and 6 months under laboratory conditions. We assessed the susceptibility of the samples to the growth of microorganisms, as well as the mass loss of the polymeric materials after composting. The findings indicated that the bioadditives increased the compostability of the materials, as evidenced by the elevated carbonyl indices observed for the samples containing biofillers. Furthermore, the elevated polar component of the surface energy exhibited by the samples containing biofillers suggested a heightened susceptibility to composting processes, attributable to their augmented hydrophilicity, in comparison to the reference EN. Fruit pomace is a promising additive for increasing the compostability of synthetic polymeric materials.

## 1. Introduction

Environmental pollution from non-degradable, everyday polymers and the need for environmentally friendly polymer materials are global challenges. With increasing ecological awareness, there has been a growing interest in materials based on biodegradable polymers made using additives of natural origin (including fillers) for several years [1,2]. However, the widespread use of biodegradable polymers such as polylactide (PLA) and thermoplastic starch (TPS) is still limited by the technical difficulty of their production and processing, making them significantly more expensive than petrochemical plastics [3,4]. Therefore, biofillers, which allow a reduction in the consumption of polymeric materials and yield compositions with unique properties, are increasingly being used and developed in polymer processing. This approach provides tangible benefits both in the processing of biopolymers by reducing the price of materials and expanding the range of their applications and in the processing of petrochemical polymers by decreasing environmental pollution [3,5,6]. The application of waste-derived natural biofillers in the processing of polymer materials offers many benefits, including reduced use of petrochemical polymers [7], faster degradation under environmental conditions [8,9], lower prices [10], reduced density [11], and reduced tribological wear on processing tools [11,12]. Furthermore, the use of specific types of biofillers can yield polymer compositions with exceptional properties, such as increased dielectric strength [13] and reduced oxygen permeability [14].

Biofilled polymer composites for synthetic polyethylene have attracted scientific interest in developing sustainable equivalents to plastics [15]. The scientific literature describes hybrid materials based on synthetic matrices such as low-density polyethylene (LDPE) [16], linear low-density polyethylene (LLDPE) [17], and high-density polyethylene (HDPE) [18] and isotactic polypropylene (iPP) [19] with natural fillers. The advantage of this concept is the use of agricultural waste, such as wood flour, cellulose, flax straw, hemp, banana, maize fiber, etc., as plant-based fillers [20]. There is growing interest in the application of food production waste as polymer additives [21,22,23]. Merino et al. [24] described polylactide-based materials containing plant wastes, such as spinach stalks, tomato pomace, and cocoa shells. Biofillers influence the biodegradability of polymer compositions. Olive pomace has been proposed as a natural filler for synthetic polyamide 6 (PA6), influencing the structure and thermal properties of the polymer [25]. The research on the application of food production waste in polymers is developing and still requires further extension because agro-wastes have proven to be multifunctional materials that can act as both stabilizers [21] and fillers [26,27]. Many studies have also confirmed that the addition of natural fillers to biocomposites accelerates their degradation [28,29].

The aim of this study was to analyze the effect of waste biofillers on the degradation of synthetic ethylene–norbornene (EN) copolymer compositions under laboratory composting conditions. Apple and chokeberry pomace at 5, 10, and 15 phr were introduced into the EN to obtain a more compostable material. So far, the literature has only described the use of waste apple peel as a reinforcing filler, with the peel improving the mechanical properties of compositions based on poly(butylene succinate) (PBS), but the corresponding publication did not reveal the effect of this filler on the biodegradability of PBS [30]. In addition, a polyvinyl alcohol (PVA)-based food-packaging film containing waste apple pomace as an active antioxidant has been described [31]. Furthermore, in order to obtain a biocomposite incorporating a natural plasticizer for food packaging, chokeberry pomace was grafted with lactic acid oligomers (OLA) synthesized in situ from lactic acid (LAc) [32].

## 2. Materials and Methods

Preparation of polymer samples: The research object was an ethylene–norbornene copolymer with the trade name Topas E-140 (Topas Advanced Polymers, Raunheim, Germany). Biofillers were added to the EN copolymer: ground apple pomace and chokeberry pomace with a particle size of 0.1–0.5 mm (AG Feeding Sp. z o.o., Gdynia, Poland). Before being processed, the biofillers were dried in a dryer at 60 °C for 12 h. The copolymer granules and biofillers were mixed in a laboratory micromixer (Plasti-Corder Brabender Lab-Station, Duisburg, Germany) at 110 °C and 50 rpm for 20 min. Then, the mixtures were formed using a two-roll mill with rolls measuring 200 mm at a roll temperature of 25 °C and a friction ratio of 1:1.1 for about 30 s. The last stage consisted of pressing the sheets between the two halves of a steel mold, lined with Teflon sheets, in a hydraulic press (temperature, 160 °C; pressure, 7.5 MPa; time, 10 min). Table 1 presents the compositions of the EN copolymers incorporating biofillers.

Composting: Six “dumbbell”-shaped (a standard specimen shape designed for the determination of mechanical properties) samples of each material were used for the tests for each time interval. The “dumbbell”-shaped samples were weighed before and after composting, and their masses were averaged. The weighed “dumbbell”-shaped samples were then placed in a ceramic vessel filled with compost soil (pH 5.5–6.5). During composting, a constant temperature (30 °C) and humidity (90%) were maintained and monitored. The samples were removed from the soil after 3 and 6 months. After removing the remaining soil with a brush, the samples were dried to a constant mass, weighed, and then further analyzed. The mass loss [%] was calculated as the difference in the average mass of the samples before and after composting and expressed as a percentage.

Evaluation of the impact of fungi on polymers (methods A and B): The tests were conducted according to the PN-EN ISO 846:2019-05 standard “Plastics—Evaluation of the action of microorganisms” [33]. The research material consisted of polymer samples with dimensions of approximately 15 mm × 15 mm. The following species of mold fungi were used in the tests: *Aspergillus niger* ATCC 16404, *Paecilomyces variotii* LOCK 0525, *Chaetomium globosum* LOCK 0475, *Trichoderma viride* LOCK 0570, and *Penicillium funiculosum* LOCK 0587. These strains were obtained from the American Type Culture Collection (ATCC, Manassas, VA, USA) and the Collection of Industrial Microorganisms at the Institute of Fermentation Technology and Microbiology (LOCK, Lodz, Poland). Depending on the test variant, different microbiological media were used. In method A, a medium with an incomplete nutrient solution (NaNO_3_, 2.0 g; KH_2_PO_4_, 0.7 g; K_2_HPO_4_, 0.3 g; KCl, 0.5 g; MgSO_4_–7H_2_O, 0.01 g; agar, 20 g; water, 1000 mL, pH 6.0–6.5) was used, while a medium with a complete nutrient solution the same as the composition given above supplemented with 30 g of glucose was used for method B. Method A, developed to test the resistance of the materials, was used to assess the natural resistance of the tested material in the absence of any other nutrients, as well as whether the tested material was a source of carbon for microorganisms. The tested material was placed on a deficient medium in Petri dishes (without a carbon source). Then, a suspension of microorganisms (10^6^ spores in 1 mL) was evenly applied to the surface of the medium and material samples. The samples were incubated at 28 °C and a relative air humidity of 80% RH for 3 months. Method B was used to determine the fungistatic effect and the influence of surface contamination on resistance. This method is used in the case of expected surface contamination. It is used to evaluate the fungistatic properties of the tested material and the influence of surface contamination on the material on its resistance. Samples of the tested material were placed on a full-value medium. Then, a suspension of microorganisms was evenly applied. The samples were incubated at 28 °C and a relative air humidity of 80% for 1 month. Subsequently, we assessed the growth of microorganisms on the surfaces of the medium and samples and checked whether any growth inhibition zones could be observed. In methods A and B, the samples were evaluated macroscopically using a grading scale according to the PN-EN ISO 846:2019-05 standard [33].

The intensity of the growth of the microorganisms on both the nutrient media and the samples was observed and evaluated using an assessment scale ranging from 0 to 5 in accordance with ISO 846 [33]: no visible growth under the microscope (0); visible growth under a microscope (1); growth covering up to 25% of the sample area (2); growth covering up to 50% of the sample area (3); significant growth, covering more than 50% of the sample area (4); and intensive growth covering the entire surface of the sample (5).

The authors presented the details of the methodology for determination in the following publication: [34].

Carbonyl Index (CI): By leveraging the Fourier transform infrared (FTIR) spectrum, we calculated the Carbonyl Index (CI), which is a measure of the number of carbonyl groups formed during the composting process, according to Equation (1):

(1) $$Carbonyl\;Index\left(CI\right)=\frac{I_{C=O}}{I_{C-H}}$$

where I_C=O_ denotes the peak intensity characteristic of C=O groups (~1700 cm^−1^), and I_C–H_ is the peak intensity corresponding to -CH groups (~2800 cm^−1^).

FTIR measurements were performed using reference and post-composting samples. Spectra were recorded in the 4000–400 cm^−1^ range using a Thermo Scientific Nicolet 6700 FTIR spectrometer equipped with a Smart Orbit ATR diamond accessory (Thermo Fisher Scientific, Waltham, MA, USA).

Determination of aging coefficient (K): Aging factors (K) were determined based on the changes in the static mechanical properties of the EN samples after composting. The mechanical properties of the materials, such as tensile strengths (TS) and elongation at break (Eb), were tested using a Zwick Roell 1435 (Zwick Roell Polska Sp.zoo. Sp.k., Wroclaw, Poland). “Dumbbell”-shaped specimens with a thickness of about 1.5 mm and a width of the middle part measuring 4 mm were prepared for testing. The following parameters were used during the determination: a tensile speed of 500 mm/min and an initial force of 0.1 N. Then, by applying Equation (2), the aging coefficients K were calculated as follows:

(2) $$K=\frac{{\left(TS\times Eb\right)}_{after\;composting}}{{\left(TS\times Eb\right)}_{before\;composting}}$$

where TS [MPa] is the tensile strength, and Eb [%] is the elongation at break.

Determination of surface free energy: This study was carried out using an OEC 15EC goniometer (DataPhysics Instruments GmbH, Filderstadt, Germany). The determination process began by measuring the wetting angles of the samples for liquids of different polarities: distilled water, ethylene glycol, and diiodomethane. Then, using SCA 20 software, surface free energy was calculated according to the Owens, Wendt, Rabel, and Kaelble (OWRK) method. The values of surface energy and surface tension in the polar and dispersed systems were summed, which allowed us to obtain Equations (3) and (4):

(3) $$\sigma_{l}=\sigma_{l}^{d}+\sigma_{l}^{p}$$

(4) $$\sigma_{S}=\sigma_{S}^{d}+\sigma_{S}^{p}$$

where $\sigma_{l}^{d}$ and $\sigma_{l}^{p}$ represent the disperse and polar parts of the liquid, respectively, whereas $\sigma_{s}^{d}$ and $\sigma_{s}^{p}$ stand for the respective contributions of the solid.

Color change after composting: The change in the color of the polymer compositions after composting was examined using a CIE-Lab system (L—lightness, a—red–green, and b—yellow–blue) using a Konica Minolta UV-VIS CM-36001 spectrophotometer (Konica Minolta Sensing, Osaka, Japan). Spectrophotometric color measurements were performed at three measurement points for each sample, and the results were averaged. Initially, the color of the samples was measured prior to the composting process. Subsequently, the change in the color of the samples was measured after composting. The change in the color of the composted samples was determined in relation to that of the standard, unaged samples. The color difference values (dE × ab) (5) were calculated using the following equation:

(5) $$dE\times ab=\sqrt{{\left(\Delta a\right)}^{2}+{\left(\Delta b\right)}^{2}{+\left(\Delta L\right)}^{2}}$$

Scanning electron microscopy (SEM) of EN compositions incorporating biofillers: The morphologies of the samples of the reference EN and the EN containing biofillers were evaluated based on photographs obtained using a scanning electron microscope (SEM) (LEO 1530 (Carl Zeiss AG, Oberchoken, Germany). The photographs were captured at a magnification of 10,000×.

## 3. Results and Discussion

**Figure 1:**
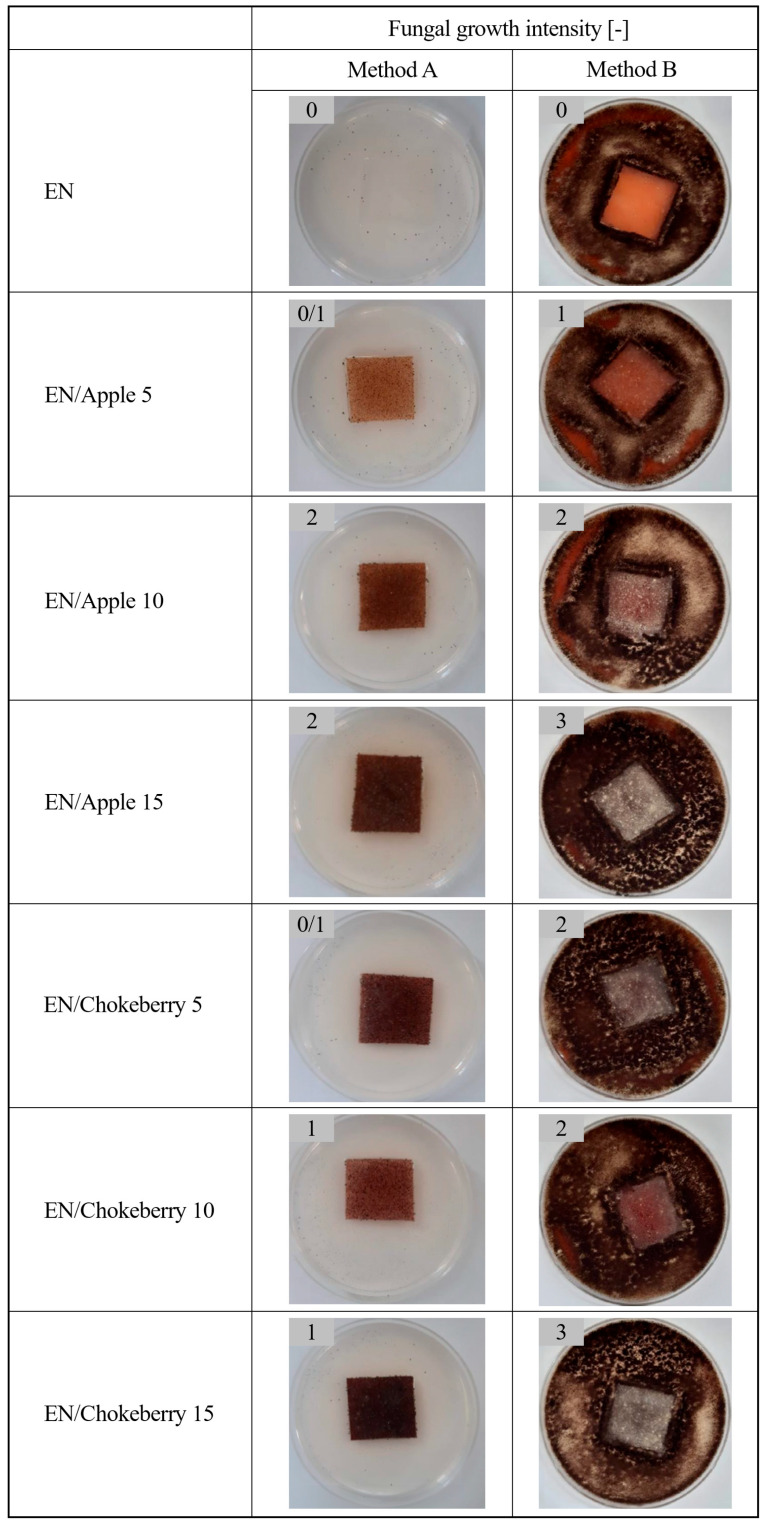
Growth intensity of filamentous fungi on EN compositions (method A—without an additional carbon source, and method B—in the presence of a microbiological medium).

This research began with an assessment of the effect of fungi (Figure 1) on polymer compositions containing biofillers, which were prepared according to the methodology presented in Section 2. Method A was a variant of the experiment in which no additional carbon source was added to the medium. In this experiment, we aimed to assess whether the test material could serve as a medium for fungi and to examine how the compounds within the polymers would interact with the test microorganisms. The aim of Method B, on the other hand, was to determine whether mold could grow on the test materials when a carbon source (in the form of a microbial medium) was present. The conditions used in the experiment are similar to those found in nature, wherein dust particles are deposited on surfaces, carrying carbon compounds with them.

The control samples of the ethylene–norbornene copolymer were not susceptible to the growth of filamentous fungi: in method A, the samples did not provide a medium for the fungi, and method B had a fungistatic effect (i.e., fungal growth intensity was 0). The incorporation of apple and chokeberry pomace additives into the materials resulted in the colonization of the samples by filamentous fungi and provided a medium for microbes (method A). Furthermore, under conditions in which access to a carbon source was granted, the addition of pomace eliminated the fungistatic effect of the materials (method B). The EN samples containing apple and chokeberry pomace were susceptible to the growth of filamentous fungi under conditions with and without an additional carbon source, indicating that they may be susceptible to biodegradation. Upon increasing the concentrations of the bioadditives, a greater intensity of fungal growth was observed (mainly for method B), which may indicate an increased susceptibility to composting.

**Figure 2:**
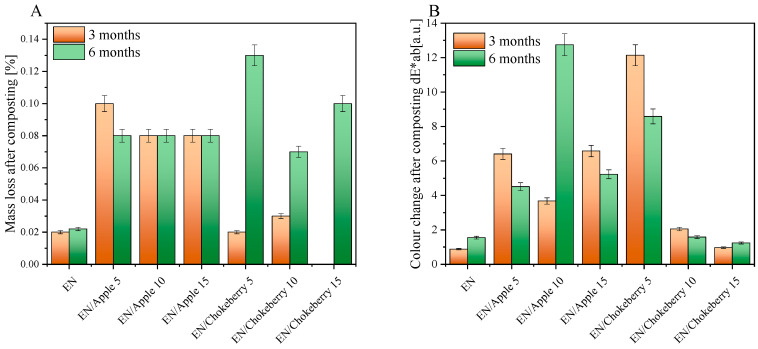
Mass loss (**A**) and color change (**B**) of the EN composition containing apple and chokeberry pomace after 3 and 6 months of composting.

In the next step, the mass losses of the samples were examined after 3 and 6 months of composting. The study results are presented in Figure 2A. For the EN reference sample, the average weight loss after 3 and 6 months of composting was not significant. In the samples that included apple pomace, the highest weight loss recorded was 0.10%. This occurred for the sample containing 5 phr of biofiller, which was composted for 3 months. For the other materials supplemented with apple waste, the weight loss was about 0.08% regardless of the bioadditive concentration and composting time. Among the samples incorporating chokeberry pomace, the greatest weight loss (0.13%) was recorded for the composition containing 5 phr of bioadditive, composted for 6 months. Analyzing the weight loss results after 3 months of composting reveals that the samples either lost a small percentage of weight or did not lose it at all. However, after further composting, the weight loss was greater for all materials containing chokeberry waste. The results obtained may be attributed to the excellent barrier properties of the EN copolymer [35], which prevented the bioadditives from encountering water and microorganisms during the composting process. As a result, the bioadditives were effectively embedded within the elastomer mass.

**Figure 3:**
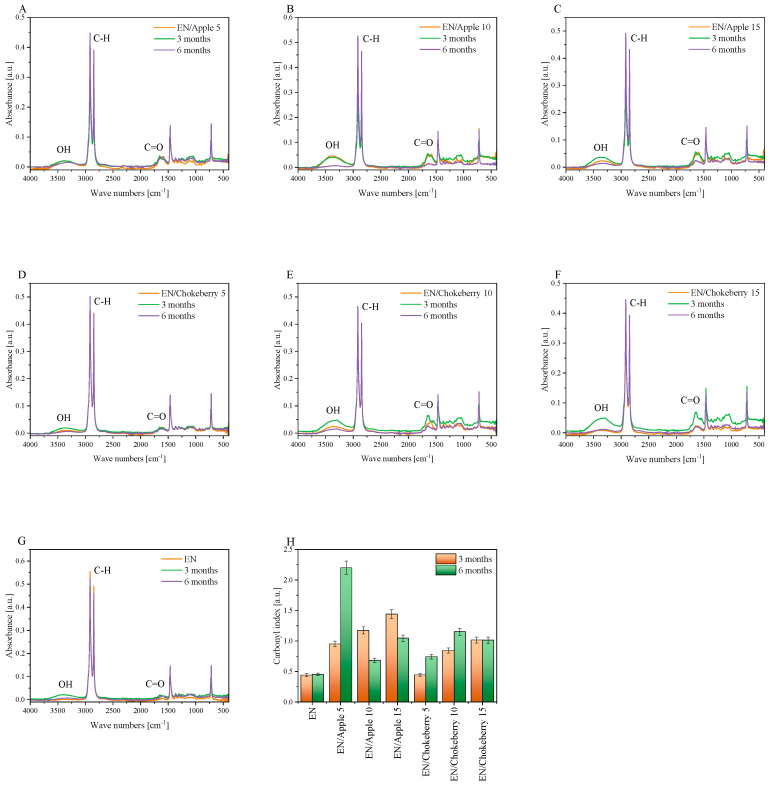
FTIR spectra (**A**–**G**) and carbonyl index (**H**) of the EN samples with biofillers after composting.

The composting of the EN samples with biofillers was accompanied by a color change (Figure 2B). A change in the color of polymeric materials can be the first sign that degradation processes are taking place in compositions. The color change factor dE × ab determined using the CIE-Lab color system mathematically describes the change in the color of polymeric materials. When the color change factor takes on values of 1 < dE × ab < 2, the difference in color can only be noticed by an experienced observer; this is the case for the EN reference sample and the composition containing chokeberry at 10 and 15 phr. The EN/Chokeberry 5 sample and the apple pomace compositions were characterized by color change factors in the range of 3.5 < dE × ab < 5.0 or above a value of 5 [-], which can be statistically interpreted as a ‘clear color difference’ and an instance wherein ‘colors can be perceived as completely different’, respectively. The greater change in the color of the samples containing bioadditives can be attributed to the oxidation of the phytochemicals they contain, a process accompanied by a change in their color. The change in the color of the materials can be correlated with the carbonyl indices determined based on infrared spectroscopy spectra (Figure 3H). The reference sample of ethylene–norbornene copolymer exhibited a slight color change, which corresponded to the lowest carbonyl indices. This may indicate the lowest oxidation of the sample surface.

Carbonyl indices were calculated based on structural changes recorded in FTIR spectra (Figure 3A–G). They can serve as a measure of the progression of the degradation of polymeric materials, as they determine the concentration of carbonyl groups (acids, aldehydes, and ketones) in a sample. FTIR analysis revealed characteristic peaks belonging to the ethylene–norbornene copolymer at 2916 cm^−1^ and 2846 cm^−1^, attributed to the stretching vibrations of the CH_2_ groups; at 1700 cm^−1^, corresponding to the carbonyl groups (C=O); at 1467 cm^−1^, indicating the CH_2_ groups (the bending band); and at 722 cm^−1^, which is the CH_2_ rocking band [36]. After composting the EN samples with apple and chokeberry, a change in the intensity of the carbonyl groups (around 1700 cm^−1^), as well as the appearance of hydroxyl groups in the range of 3500–3000 cm^−1^, was observed. This new OH band may indicate the degradation of polymeric materials with bioadditives. The degradation of polymer composites can be assessed by determining the carbonyl index since various carbonyl products are formed during degradation, including carboxylic acids (1705–1712 cm^−1^), ketones (1715–1725 cm^−1^), esters (1735–1750 cm^−1^), and lactones (1760–1785 cm^−1^) [37]. The carbonyl indices were calculated by considering the peak intensities at approximately 1700 cm^−1^ (carbonyl groups) and approximately 2800 cm^−1^ (-CH groups). Figure 3H summarizes the values of the carbonyl indices after composting the EN composition. An increase in this index indicates a greater intensity of the degradation process during composting. The reference EN sample was characterized by the smallest change in carbonyl indices: for the sample subjected to 3 and 6 months of composting, the CI was about 0.5 [-]. In the case of CI composting, the reference samples were similar after composting over the respective time intervals. However, for the materials with pomace, the CI was slightly higher, which may indicate that the bioadditives increased the degradability of the synthetic samples. Similar results were obtained for the EN composite supplemented with the organic filler cellulose, which is much more susceptible to oxidation processes during photo-oxidation than the reference polyolefin [37]. Moreover, a tendency toward a faster degradation of materials containing cellulose has also been observed in the case of polypropylene-based products [38,39]. For the samples containing pomace incorporating chokeberry, the CI index increased with composting time, indicating greater structural changes in the materials. The greatest structural changes (the greatest CI) were exhibited by the samples supplemented with apple biofiller. The ambiguous trend in the values of the CI indices in the composting time intervals may be due to the inhomogeneity of the materials.

As indicated in the literature [40], the biodegradation mechanisms of polymers are contingent upon their chemical structures, which can be categorized as either heterochain or carbon backbone. The degradation mechanism of heterochain backbone polymers is based on chemical degradation through hydrolysis or enzyme-catalyzed hydrolysis [41]. The hydrolytic biodegradation process is contingent on the hydrolytic enzymes secreted by microorganisms and the physicochemical properties of the polymer in question. It has been demonstrated that the biodegradation of a polymer can occur within a period of one month as a consequence of hydrolysis [42]. Hydrolytic biodegradation has been observed in a number of naturally occurring biopolymers, including polysaccharides and proteins, plant-derived polymers such as polylactide, and microbiologically synthesized polymers [41]. A variety of factors have been identified as potential influences on the rate of hydrolytic degradation, including chemical bonding, the type of copolymer, thickness, water absorption, and sample morphology. However, the biodegradation of heterochain and carbon-chain polymers and carbon backbone polymers is slow [41]. The degradation of carbon backbone polymers involves chemical degradation through oxidation or enzyme-mediated oxidative degradation. It has been established that a number of naturally occurring biopolymers, including lignin, are subject to the process of oxidative biodegradation. The oxidative biodegradation of polymers can take years, far exceeding the duration of hydrolytic biodegradation.

In the context of ethylene–norbornene copolymer composites mixed with fruit pomace, a range of biodegradation mechanisms may be observed. Agro-waste serves as a source of various natural biopolymers, which exhibit diverse decomposition mechanisms. Chokeberry pomace has been found to contain mono- and polysaccharides [43], which are hydrolytically biodegradable [40]. Moreover, the composition of apple and chokeberry agro-waste is distinguished by the presence of plant fibers. In [44], the lignin, cellulose, and hemicellulose contents of chokeberry pomace were found to be 32.76%, 18.87%, and 3.53%, respectively, while the lignin, cellulose, and hemicellulose contents of apple pomace were found to be 9.46%, 20.99%, and 5.87%, respectively. The authors of [44] highlighted that the analysis of lignocellulose content in fruit pomace yielded results different from those of other published studies, suggesting the heterogeneous nature and diversity of apple and chokeberry byproducts. The lignin in the pomace can decompose via oxidative biodegradation, and the degradation of natural polymers can take much longer than hydrolytic biodegradation [41]. The new hydroxyl groups, observable in the 3500–3000 cm^−1^ range in the FTIR spectra of the EN samples containing fruit pomace (Figure 3A–F), may be indicative of the hydrolytic or oxidative degradation of the bioadditives.

According to the available literature [45] on physico-chemical aging (degrading factors: temperature, UV, and ionizing radiation), the degradation of the polymer matrix, i.e., the ethylene–norbornene copolymer, most often takes place through oxidation. FTIR spectra [45] showed the occurrence of specific functional groups, namely (C=C) and (C=O) unsaturated bonds (vinyls, α-β-unsaturated ketones, esters, acids, ketones, lactones, etc.), associated with the degradation processes of ethylene–norbornene copolymers. In general, oxidation was identified as the most pronounced degradation process for EN copolymers when norbornene yields increased, as amorphous regions became more frequent and facilitated oxygen diffusion. In the majority of the cases described [45], physicochemical aging caused chain scission, which led to the formation of degradation compounds that showed high mobility, corresponding to their low molecular weight and/or high polarity. Furthermore, it is conceivable that crosslinking may have occurred during the aging process, resulting in a modification of the mechanical properties of EN materials [45].

**Figure 4:**
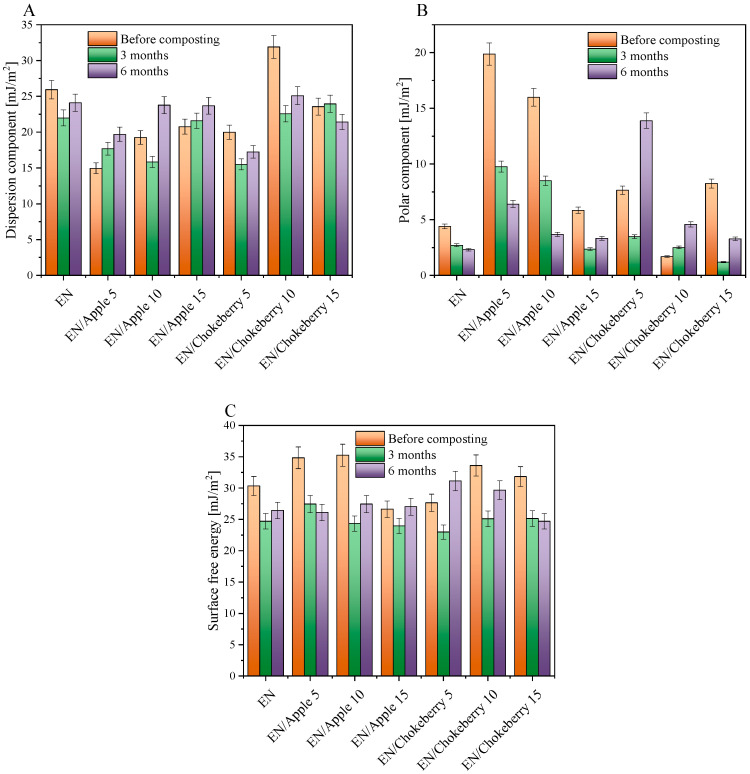
Dispersive (**A**) and polar (**B**) components representing the sum of surface free energy (**C**) for the EN samples with biofillers before and after composting.

**Figure 5:**
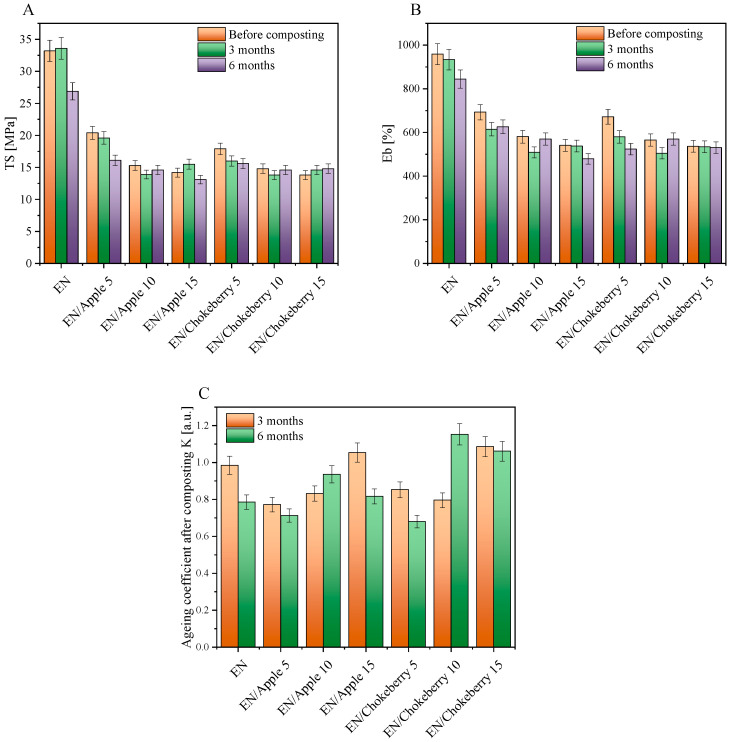
Mechanical properties ((**A**) TS—tensile strength; (**B**) Eb—elongation at break) and aging coefficients K (**C**) of EN samples with biofillers after composting.

For the EN compositions supplemented with chokeberry and apple pomace, the predominant degradation mechanism for the polymer matrix may have been the oxidation of ethylene–norbornene copolymers, while the biofillers may have undergone both oxidative and hydrolytic biodegradation. The addition of biofillers increased the hydrophilic character (polarity) of the samples (Figure 4B), and, thus, the biodegradation mechanisms of the composites may have initially been related to the hydrolytic biodegradation of the natural additives, which can be degraded more easily than the polymer matrix. Subsequently, the decomposition of the biofillers may have increased the diffusion of oxygen deep into the samples, and the oxidative degradation of the materials and a slow breakdown of the polymer chains may have occurred. Such slow decomposition of the polymer chains could be evidenced by the gradual change in the mechanical properties of the composites (Figure 5A,B)—i.e., a decrease in the values of the tensile strength and elongation-at-break parameters.

However, the biodegradability of lignocellulosic composites is contingent on numerous factors, including the polymer matrix, the composition of lignocellulosic fibers utilized as fillers, and environmental factors such as moisture, humidity, temperature, and microbial populations. The rate of biodegradation of lignocellulosic composites is affected by environmental factors [40].

In order to determine the surface properties of the samples, we determined their surface free energy and its polar and dispersion components (Figure 4). The surface free energy of the samples was determined by calculating the sum of the polar and dispersion components. The polar component is defined as the sum of the hydrogen, acid–base, and inductive forces, while the dispersion component determines the magnitude of intermolecular interactions known as London forces. The polar component of surface energy is indicative of the susceptibility of polymeric materials to oxidative processes. For the reference EN, the polar component was 4.4 mJ/m^2^ before composting. The addition of biofillers increased the polar components of most of the samples (except for EN/Chokeberry 10) by about 1.5 to 15.5 mJ/m^2^. Higher polar component values might indicate a more hydrophilic nature of the composition—associated with the addition of a hydrophilic filler—and therefore a potentially greater susceptibility of the materials to composting. Biofillers from waste apples and chokeberry contain substances of a hydrophilic nature, such as cellulosic fiber [46], as well as polyphenolic compounds [47,48] present in the fruits. The hydrophilic nature of biofillers has a favorable impact on the compostability of polymeric composites, e.g., by increasing the possible hydrolytic biodegradation. Nevertheless, plant additives are characterized by a unique composition, and the uneven distribution of bioadditives could have influenced the lack of trend in the surface energy results before and after composting.

After 3 months of composting, decreases or slight increases in the polar component (Figure 4B) and surface free energy (Figure 4C) values were found for the samples tested, indicating little or no degradation. In the case of composting lasting 6 months, only the EN samples containing 5 phr and 10 phr of chokeberry pomace had higher polar component and higher or comparable surface free energy values. Such a change after a longer composting time may indicate slow progress of the degradation process. The greatest progress in degradation during composting was observed in the EN/Chokeberry 5 phr sample, for which, after 6 months of composting, the polar component and surface energy were 6.24 mJ/m^2^ and 3.48 mJ/m^2^ higher than those before composting.

The ambiguous results regarding the determination of the surface energy and its components could be a product of the non-uniform composition and distribution of bioadditives in the materials, as well as the presence of moisture that was absorbed by the samples during composting. Prior to the measurement, the samples were subjected to a drying procedure. However, it is plausible that this procedure did not facilitate the complete elimination of moisture, particularly that which had been absorbed into the internal structure of the biofillers, as well as moisture stored on the surfaces of the samples.

**Figure 6:**
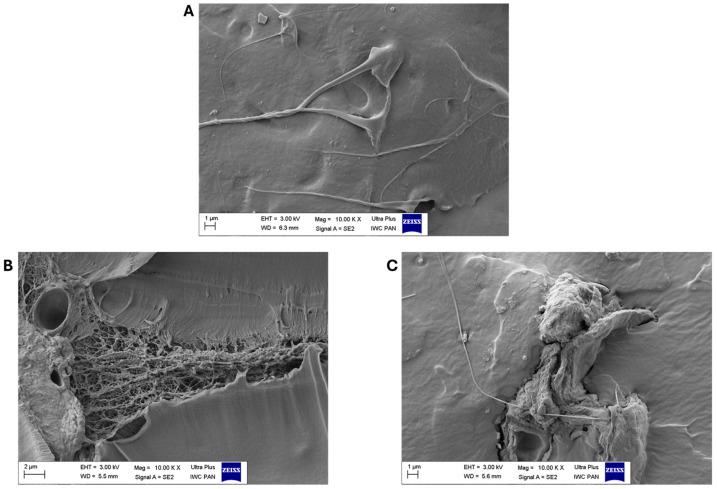
SEM photos of compositions EN (**A**), EN/Apple 10 (**B**), and EN/Chokeberry 10 (**C**) at 10,000× magnification.

Figure 5 shows the mechanical properties of the EN compositions and the aging factors calculated from them after composting. The addition of biofillers significantly influenced the mechanical properties of the ethylene–norbornene copolymers. The reference samples filled with the greatest amounts of apple and chokeberry pomace exhibited mechanical properties that were over 50% weaker than those of the standard EN (the tensile strength (TS) parameters were as follows: EN—33.2 MPa, EN/Apple 15—14.2 MPa, and EN/Chokeberry 15—13.8 MPa). The most-filled samples also exhibited the lowest elongation-at-break values (the Eb values for individual samples were as follows: EN—959%, EN/Apple 15—541%, and EN/Chokeberry 15—537%). We hypothesize that the materials’ weaker mechanical properties may have been due to their inhomogeneity and the pronounced presence of inclusions of plant material fragments. The SEM images in Figure 6 show the morphologies of the reference EN sample (A) and compositions containing 10 phr of fruit pomace (B,C). In comparison with the smooth reference ethylene–norbornene copolymer, the samples with biofillers are characterized by increased porosity, as well as a pronounced presence of bioadditive inclusions. The greater number of pores observed in the materials containing apple and chokeberry pomace has been shown to lead to structural discontinuity and the deterioration of stress transfer mechanisms, thus reducing the tensile strength of materials [49]. Conversely, the elongation at break of polymer compositions, in addition to porosity, can also be affected by insufficient interfacial adhesion. This phenomenon, in the case of composites containing apple and chokeberry agro-waste, can be attributed to the chemical composition of the bioadditives and the differences in polarity between the hydrophilic components of the biofillers and the hydrophobic polymer matrix [50].

Composting had little effect on the samples, and they retained an average tensile strength and elongation at break of 98% and 92%, respectively, relative to the samples before aging. The aging factor indicates the resistance of the samples to degradation. K values close to 1 [-] mean that a material is resistant to degradation, while K values close to 0 [-] indicate the susceptibility of samples to degradation. The aging coefficients of the EN sample were 0.985 [-] and 0.786 [-] after 3 and 6 months of composting, respectively. The results therefore indicate that the compostability of the EN copolymer increased slightly with time. For the materials containing apple and chokeberry pomace, no clear trend in the change in K-factors was found. For EN/Apple 5 and 15 and EN/Chokeberry 5, an increase in the susceptibility of the samples to degradation was observed with an increase in composting time (with lower K-coefficient values after 6 months of composting compared to those after 3 months). For the other materials, K-factor values that were similar or slightly higher were recorded after 6 months of composting. This phenomenon may be due to the inhomogeneous dispersion of the biofillers in the samples and the natural heterogeneity in the composition of the fruit pomace, i.e., the different content of cellulose fibers and polyphenolic compounds remaining in the individual samples, whose presence may have influenced the achievement of higher TS and Eb values during the mechanical properties test.

## 4. Conclusions

Microbiological evaluation and analysis of the weight changes of the compositions after 3 and 6 months of composting showed that the addition of apple and chokeberry pomace promoted the growth of filamentous fungi and led to greater weight loss of the samples in comparison to the reference ethylene–norbornene copolymer. Furthermore, surface analysis of the samples confirmed that greater structural changes (in relation to carbonyl indices) occurred for the materials containing biofillers, and higher values of the polar component of the surface energy indicated a potentially greater susceptibility of the samples to composting processes, due to greater hydrophilicity, compared to standard EN. The more pronounced color change in most of the EN samples supplemented with waste additives also suggests a greater susceptibility of these materials to degradation processes. Plant additives have shown promise in terms of increasing the compostability of synthesized EN samples. To achieve a higher degree of compostability of ethylene–norbornene copolymer-based materials, researchers should consider introducing more biofillers into the composition or modifying the extrudates to improve compatibility with the polymer matrix (thereby enabling better homogeneity of the materials). Time and process conditions will also have a significant impact on the compostability of materials containing biofiller. The use of fruit pomace generated during food production may prove to be way of utilizing agricultural waste as biofillers for synthetic polymeric materials, enabling increased degradability of polymer compositions.

### Future Prospects

The research results obtained provide valuable data on the optimization of the composition of EN compositions containing pomace. We hypothesize that an increase in the amount of biofiller additives above 15 phr will result in a more favorable outcome with regard to composting materials. However, it is important to note that the addition of more agro-waste may result in a weakening of the mechanical properties of the samples. Therefore, when designing the optimal composition of EN composites, it is essential to take the expected strength of the materials into account. Furthermore, in order to increase the compostability of samples based on EN and agro-waste, it is necessary to consider the use of more aggressive composting conditions, such as industrial composting, characterized by an elevated temperature (e.g., about 50–70 °C), appropriate humidity and amounts of oxygen, and suitable quantities and types of microorganisms.

## Figures and Tables

**Table 1:** The compositions of ethylene–norbornene (EN) copolymers containing biofillers (apple pomace and chokeberry pomace). The compositions of the samples prepared are given in parts per hundred rubber (phr).

	EN	Apple	Chokeberry
EN	100	-	-
EN/Apple 5	100	5	-
EN/Apple 10	100	10	-
EN/Apple 15	100	15	-
EN/Chokeberry 5	100	-	5
EN/Chokeberry 10	100	-	10
EN/Chokeberry 15	100	-	15

## Data Availability

Dataset available on request from the authors.
